# Casual blood pressure and ambulatory blood pressure measurement in children

**DOI:** 10.1590/S1516-31802003000200011

**Published:** 2003-03-05

**Authors:** Vera Hermina Koch

**Keywords:** Blood, Pressure, Methodology, Children, Adolescent, Pressão, Arterial, Metodologia, Criança, Adolescente

## Abstract

Some epidemiological studies on blood pressure among children and adolescents have revealed that blood pressure levels in childhood are the strongest predictors of adult blood pressure levels. In the adult population, hypertension causes a two to threefold increase in an individual's risk of cardiovascular morbidity. Cardiovascular risk depends on blood pressure itself, coexistent risk factors and whether there is hypertensive end-organ damage. Therefore, accuracy in determining blood pressure is essential and a standardized protocol should be considered for blood pressure measurement, which would make the comparison of results obtained by different studies in different countries possible. This article reviews the main determinants of accuracy for casual and ambulatory blood pressure measurements in children.

## RESUMO

Estudos epidemiológicos envolvendo medida de pressão arterial em crianças e adolescentes têm demonstrado que o valor da medida de pressão arterial na infância constituise no maior preditor dos níveis pressóricos do adulto. A hipertensão arterial no adulto eleva em duas a três vezes o risco individual de desenvolvimento de morbidade cardiovascular. O risco cardiovascular depende da pressão arterial propriamente dita, de fatores coexistentes e da presença de lesão instalada de órgãos-alvo. A acurácia na determinação da pressão arterial é, portanto, mandatória e um protocolo estruturado e padronizado para sua obtenção deveria ser estabelecido, possibilitando a comparação de resultados de estudos realizados em diferentes países. Este artigo discute os maiores determinantes da precisão de medida da pressão arterial casual e ambulatorial na criança.

Cardiovascular diseases are the main causes of death in Brazil. Stroke mortality rates among Brazilians are high, reflecting the burden of hypertension. Some international epidemiological studies on blood pressure among children and adolescents have revealed that blood pressure levels in childhood are the strongest predictor of adult blood pressure levels.^1-3^ In the adult population, hypertension causes a two to threefold increase in an individual's risk of cardiovascular morbidity.^4,5^ The relationship between hypertension and cardiovascular disease seems to be continuous: cardiovascular risk depends on blood pressure itself, coexistent risk factors and whether there is hypertensive end-organ damage.

As accuracy in determining blood pressure is essential, a standardized protocol should be considered for blood pressure measurement, which would make the comparison of results obtained by different studies in different countries possible. Observers should be trained and certified to minimize measurement bias. Homogeneous decisions should be taken regarding equipment factors such as an appropriate cuff bladder size or the alternative use of mercury manometers or oscillometric devices. Technical factors such as the recording of fourth, fifth or both Korotkoff sounds for diastolic blood pressure need to be taken into consideration. Also, the number of measurements needed for estimating a child's blood pressure and the influence on its measured value of environmental factors such as the time of the day and ambient temperature must be considered.^6^ Some of these factors will be discussed separately in the next paragraphs.

## The cuff

Classically, to obtain an accurate blood pressure measurement, a cuff bladder width of approximately 40% of the upper arm circumference should be chosen because it most closely approximates intra-arterial readings.^7^ The bladder length should be at least 90% of arm circumference to avoid overestimation of blood pressure, especially in children.^8^ Another less-known effect of the cuff size change occurs when, in accordance with the abovementioned instructions for cuff selection, the cuff size is changed to a larger one. In this case, the cuff change leads to an abrupt fall in the value of measured blood pressure that is not arm-dependent, but cuff-dependent.^9^ This very inconvenient effect may be responsible for two issues: 1. Any association between blood pressure and arm circumference, such as body mass, will be biased towards zero. 2. In longitudinal studies, when changing to a larger cuff, measured blood pressure is lower than previous readings, which could lead to inappropriate inverse correlations of blood pressure with chronological age or height. In 1999, Arafat and Mattoo^10^ reviewed commercially available blood pressure cuffs and detected that the sizes of available cuffs, labeled as infant, pediatric, small adult, adult and large adult were heterogeneous among the different manufacturers. These authors concluded that cuff sizes need to be standardized and indicate bladder size, and suggested that they should be color-coded for convenience.

## Number of measurements needed

Another important issue to consider is the number of measurements that should be repeated within a visit and between visits in order to determine a child's blood pressure. The work by Gillman and Cook (1993)^6^ demonstrated that it depends on the instrument and technique. For auscultatory equipment, using a mercury manometer or random zero manometer, among 162 children aged 8 to 12 years, the systolic blood pressure values obtained after four weekly visits with three measurements per visit leveled off after about 2-3 measurements per visit, but the difference between visits was large until about the third or fourth visit. For oscillometric equipment, using the Dinamap model 845XT, among 106 children aged 9 to 13 years, the systolic blood pressure values obtained after three weekly visits with four measurements per visit demonstrated that for the Dinamap device the first of several measurements during one particular visit was generally higher than the following ones. The values obtained started to level off after 4-5 measurements within a visit, with the "first measurement effect" reproducible even after 3 consecutive visits.

## The diastolic dilemma

There has been an ongoing controversy over whether the muffling (Korotkoff 4-K4) or disappearance of sounds (Korotkoff 5- K5) should be preferentially considered for the measurement of diastolic blood pressure in children.^11^ Neither value correctly defines intra-arterial diastolic blood pressure, since K5 is approximately 9 mmHg higher than direct diastolic blood pressure and K5 is easier for the human ear to discern than K4^12^. Current recommendations therefore favor the use of K5.

## The stethoscope diaphragm versus the bell

The bell is preferred for blood pressure auscultation in adults. This issue is still controversial in children, since placing the bell adequately in small children may compress the artery and produce falsely low diastolic values. Thus, some authors advocate the use of the diaphragm for small children,^13^ while others suggest that the bell, when properly used, should accomplish better auscultatory results.^11^

## Time of the day and ambient temperature

It is clear from ambulatory blood pressure studies that blood pressure varies over the 24 hours of the day, presenting lower values during sleep and higher values during wakefulness, with a peak in the morning and another in late afternoon.^14^ There is a negative relationship between blood pressure and temperature. An increase of 10°C leads to a fall of approximately 5-7 mmHg in systolic and in diastolic blood pressure.^15,16^

## Do we have normative blood pressure data for children?

Unfortunately we don't have normative blood pressure data for the pediatric population. Table 1 shows the lack of homogeneous methodology in nine studies that made up the Second Task Force of Blood Pressure Measurement in Children, reviewed by Rosner et al. in 1993.^17,18^ The Update of the Second Task Force of Blood Pressure Measurement in Children added a tenth study to this list (National Health and Nutrition Examination Survey — NHANES III).^19^ Table 2 shows the same lack of methodological homogeneity in the six studies from which the European pediatric blood pressure normative data is at present derived.^20^

**Table 1:** Methodology parameters of the nine studies that made up the Second Task Force of Blood Pressure Measurement in Children^17,18^

Source	Age (years)	Instrument	Cuff width	Cuff length	Number of observers	Place of measurement
NIH	6 - 17	Mercury column	9.5 × 13	-	Multiple – physicians	Vans
Pittsburgh	1 - 5	Doppler	-	≥ 75% AC	-	Home
Dallas	13 - 17	Random zero	Multiple AC	Most of AC	Multiple	School
Bogalusa	1 - 17	Mercury column	4 cuffs AC	≥ 50% AC	3	School
Houston	3 - 17	Mercury column	2/3 arm length	≥ 75% AC	Multiple	Clinic
South Carolina	4 - 17	Mercury column	Multiple AC	-	Multiple	School
Iowa	5 - 17	Doppler Random zero	4 cuffs	-	Multiple	School
Providence	1 - 3	Random zero	2/3 arm length	-	Multiple	Clinic
Minnesota	9 - 17	Mercury column	5 cuffs AC	≥ 90% AC	4	School

*NIH: National Institutes of Health; AC: arm circumference.*

**Table 2:** Methodology parameters for the six studies that made up the European pediatric blood pressure normative data^19^

Source	Age (years)	Instrument	Cuff (cm)	Position	Place of measurement
**Berlin-Bremen**	11 - 17	Random zero	9 × 18 12 × 23 14 × 28	Sitting, Right arm	school
**Cologne**	15 - 19	LSH	12.5 × 28	Sitting, Right arm	school
**Copenhagen**	6 - 18	Random zero	6 × 20 9 × 28 12 × 35	Sitting, Right arm	school
**Essen**	4 - 18	LSH	2/3 arm length 8 × 2010 × 25	Sitting, Right arm	school/open
**Nancy**	4 - 17	Mercurycolumn	2/3 arm length9 × 22 12 × 26	Supine, Left arm	open
**Zoetermeer**	5 - 19	Random zero	10 × 23 14 × 23	Sitting, Left arm	open

*LSH: London School of Hygiene equipment.*

It is important to emphasize that this lack of homogeneity is not a consequence of carelessness but rather of the multiple difficulties involved in performing epidemiological studies in the pediatric age group. Unfortunately, according to Nielsen et al. (1989)^21^, "confusion concerning the most suitable cuff… is responsible for at least some of the scatter between blood pressure studies". Arafat and Mattoo (1999),^10^ referring to the Update of the Second Task Force of Blood Pressure Measurement in Children, suggested that "a new multicenter study, using uniform criteria for cuff selection, may be necessary to establish the accuracy of the published nomogram on normal blood pressure in children".

## What blood pressure measuring device should be used in the future?

The mercury manometer is our old friend. It is simple, accurate and easy to service. Standard Hg readings are the main basis for blood pressure-disease associations and, although blood pressure readings with this instrument are subject to terminal digit preference and observer bias, observer training could possibly eliminate this problem. Unfortunately, mercury has toxic effects on the environment and the mercury manometer will have to be gradually replaced.

The aneroid sphygmomanometer registers blood pressure through a mechanically intricate system. Its accuracy is affected by everyday use. When calibrated against a mercury manometer a mean difference of 3 mmHg is acceptable, although up to 30% have errors of more than 7 mmHg. Readings are also subject to terminal digit preference and observer bias^22^.

What about automated sphygmomanometry? The most widely used oscillometric devices are manufactured under the name "Dinamap". Several models have been developed, each with an updated algorithm. Validation data has to be obtained separately for each model. Systolic and diastolic blood pressures are calculated as a function of the mean arterial pressure, which is the point of maximal oscillation and are calibrated to be equivalent to intra-aortic pressures. The devices are easy to use and strongly correlated to intra-arterial readings. Accuracy is affected by arm movement and measurements are affected by the "first-reading effect".^23^

Automated oscillometric devices have to be validated before they can be recommended for clinical use. Validation protocols based on comparative measurements between oscillometric equipment and the mercury manometer were devised by the British Hyper-tension Society and the American Association of Medical Instruments.^24^ The two protocols have now been reconciled and are used in association to validate oscillometric devices. Table 3 presents the instruments currently validated and recommended for hospital use and self-measurement (home blood pressure).^25,26^

**Table 3:** Automated oscillometric blood pressure measuring devices recommended for hospital use and self measurement (upper arm)^23^

DEVICE	AAMI	BHS	USE
**HOSPITAL USE**			
Datascope Accutorr Plus	Passed	A/A	At rest
CAS Model 9010	Passed	-	At rest (adults)In neonates
**SELF MEASUREMENT**			
Omron HEM 705 CP	Passed	B/A	At rest
Omron HEM 722 C	Passed	A/A	At rest (elderly people)
Omron HEM 735 C	Passed	B/A	At rest (elderly people)
Omron HEM 713 C	Passed	B/B	At rest
Omron HEM 737 Intellisense	Passed	B/B	At rest

*AAMI: Association for the Advancement of Medical Instruments; BHS: British Hypertension Society: A and B represent grading criteria for evaluating the devices proposed by the BHS for systolic/diastolic pressure. Criteria for fulfilling protocol are that the mean difference between the standard sphygmomanometer and the device being validated should be within < 5 mmHg (SD < 8 mmHg). Grades represent the cumulative percentage of readings in agreement with 5 mm Hg, 10 mm Hg, and 15 mm Hg of the mercury standard. Grade A denotes greatest agreement with mercury standard and D denotes least agreement. A=best agreement (recommended for clinical use); B=good agreement (recommended); C=poor agreement (not recommended); D=worst agreement (not recommended).* ^25^

Is it possible to use auscultatory and oscillometric devices interchangeably? Unfortunately not, as Korotkoff is approximately 3 mmHg lower than direct systolic blood pressure and, as we mentioned earlier, K5 is approximately 9 mmHg higher than direct diastolic blood pressure^12^. Park et al. (2001)^27^ tested the Dinamap 8100 against the standard mercury manometer and found that the equipment detected mean systolic and diastolic blood pressure values significantly above auscultatory readings. On the other hand, Barker et al. (2000)^28^ tested the Omron M1 against the standard mercury manometer and concluded that the Omron M1 overestimates higher pressures and underestimates lower pressures. There is a lack of validated and approved automated devices for use in clinical and epidemiological setting for the pediatric age group.^29^

## Ambulatory blood pressure monitoring in children

The current general indications for ambulatory blood pressure monitoring are: identification of white coat hypertension, borderline hypertension, identification of nocturnal hypertension, drug resistant hypertension, indication of antihypertensive medication, hyper-tension of pregnancy and identification of hypotension.^30^ Among the current issues for ambulatory blood pressure monitoring use in pediatrics, the main problem is the lack of definite normative data. The methodology is promising, since recordings show good accuracy and reproducibility in children.^31^ Up-to-date definitions of sleep/wake periods, using actigraphy or a detailed diary of daily activities, are necessary for accurately determining the sleep blood pressure decline.^32^ The white coat effect (white coat hypertension or white coat normotension) known within the literature relating to adults has also been confirmed in the pediatric population. In the same way as for adults, the left ventricular mass index and left ventricular hypertrophy are more closely related in children to 24-hour systolic blood pressure than with casual systolic blood pressure.^33^ According to Kapuku et al. (1999),^34^ left ventricular hypertrophy can be predicted by initial ambulatory systolic parameters.

In a recent study,^35^ our group compared casual blood pressure and ambulatory blood pressure monitoring parameters among normotensive and hypertensive adolescents. Casual blood pressure was measured by two trained observers in two different and separate environments (clinic and ambulatory blood pressure monitoring unit). For systolic and diastolic blood pressure, in both normotensive and hypertensive populations, an alarm reaction was demonstrated during exposure to an unknown environment and observer (the ambulatory blood pressure monitoring unit). It should also be noted that, contrary to findings in adult populations, the mean casual systolic/diastolic blood pressure measured in the clinic was lower than the mean ambulatory blood pressure monitoring parameters while awake, for normotensive and hypertensive adolescents. The same study compared findings from casual auscultatory measurements (in the clinic and ambulatory blood pressure monitoring unit) and ambulatory blood pressure monitoring parameters among hypertensive adolescents. The parameters included systolic and diastolic ambulatory blood pressure monitoring methods, systolic and diastolic blood pressure descent during sleep (systolic/diastolic sleep blood pressure descent), systolic and diastolic blood pressure load. This led us to conclude that, although normality parameters are still under development for ambulatory blood pressure monitoring in the pediatric age range, ambulatory blood pressure monitoring is a promising tool for the follow-up of pediatric hypertensive patients. In this respect it seems superior to casual blood pressure evaluation, since it uncovers the white coat effect.

Ambulatory blood pressure monitoring device validation data for children is scarce. The Spacelabs 90207, widely used in pediatric studies, and the TM 2421, used in a recent large pediatric study^36^ are equipment that has not scored well enough to be recommended according to the protocols of the British Hypertension Society and the American Association of Medical Instruments. At present, the only device recommended for children, according to these protocols, is the QuietTrak^37^ Amazingly, this is a piece of auscultatory equipment, a type of device generally not adopted in pediatric studies because the noise of children in movement interferes with the accuracy of the microphone determination of the measured blood pressure value. Table 4 shows a list of some large pediatric ambulatory blood pressure monitoring studies^36,38-42^ and demonstrates that, as for casual blood pressure, studies are being performed without methodological homogeneity. Different devices, with different measurement protocols, cannot be considered together to generate norms.

**Table 4:** List of some large pediatric ambulatory blood pressure monitoring studies showing methodological heterogeneity

Study	Device	Casual BP	Methodology	Interval between measurements	Data Analysis
Lurbe^32^	Spacelabs 90207	Mean of 3 mercury column	O	20 - 20’ day 30 - 30’ night	linear
Harshfied^33^	Spacelabs 5200	Mean of 10 Dinamap	O	20 - 20’ day 60 - 60’ night	linear
Accutracker II			A	20 - 20’	
Reichert^35^	Spacelabs 90207 Medilog	-	OA	15 - 15’ day 30 - 30’ night	linear
Lurbe^34^	Spacelabs 90207	Mean of 3 mercury column	O	20 - 20’ day 30 - 30’ night	Fourier
Soergel^36^	Spacelabs 90207 Meditech		OO	15 – 20’ day 30 – 50’ night	linear
O'Sullivan^37^	TM2421	Mean of 4TM2421	O/A	30 - 30’ day 60 - 60’ night	linear

*A = auscultatory ; O = oscillometric.*

In conclusion, as of today, the main problem for the diagnosis and management of hypertension in children is the lack of good normative data for casual and ambulatory blood pressure values. The only solution for this issue is to propose a multicenter study with a homogenous protocol, in order to obtain normal multiethnic casual and ambulatory pediatric blood pressure values. Only then will studies to correlate blood pressure level and hypertensive end-organ damage be possible.
